# Striatal amyloid is associated with tauopathy and memory decline in familial Alzheimer’s disease

**DOI:** 10.1186/s13195-019-0468-1

**Published:** 2019-02-04

**Authors:** Bernard J. Hanseeuw, Francisco Lopera, Reisa A. Sperling, Daniel J. Norton, Edmarie Guzman-Velez, Ana Baena, Enmanuelle Pardilla-Delgado, Aaron P. Schultz, Jennifer Gatchel, David Jin, Kewei Chen, Eric M. Reiman, Keith A. Johnson, Yakeel T. Quiroz

**Affiliations:** 1 Massachusetts General Hospital, Harvard Medical School, 149 13th Street, Suite 10.014, Charlestown, Boston, MA 02119 USA; 2 Cliniques Universitaires Saint-Luc, Université Catholique de Louvain, Brussels, Belgium; 3 Universidad de Antioquia, Medellín, Colombia; 4 Brigham and Women’s Hospital, Harvard Medical School, Boston, MA USA; 5 Banner Alzheimer Institute, Phoenix, AZ USA

**Keywords:** Early-onset autosomal dominant Alzheimer’s disease, Striatum, Tau PET, Amyloid PET, Memory

## Abstract

**Background:**

Autosomal dominant Alzheimer’s disease (ADAD) is distinguished from late-onset AD by early striatal amyloid-β deposition. To determine whether striatal Pittsburgh compound B (PiB)-PET measurements of amyloid-β can help predict disease severity in ADAD, we compared relationships of striatal and neocortical PiB-PET to age, tau-PET, and memory performance in the Colombian Presenilin 1 E280A kindred.

**Methods:**

Fourteen carriers (age = 28–42, Mini-Mental State Examination = 26–30) and 20 age-matched non-carriers were evaluated using PiB, flortaucipir (FTP; tau), and memory testing (CERAD Word List Learning). PiB-PET signal was measured in neocortical and striatal aggregates. FTP-PET signal was measured in entorhinal cortex.

**Results:**

Compared to non-carriers, mutation carriers had age-related elevations in both neocortical and striatal PiB binding. The PiB elevation in carriers was significantly greater in the striatum than in the neocortex. In mutation carriers, PiB binding in both the neocortex and the striatum is related to entorhinal FTP; however, the association was stronger with the striatum. Only striatal PiB was associated with worse memory. Remarkably, PiB binding in the striatum, but not in the neocortex, predicted entorhinal FTP and lower memory scores after adjusting for age, indicating that striatal PiB identified the carriers with the most severe disease.

**Conclusions:**

Based on these preliminary cross-sectional findings, striatal PiB-PET measurements may offer particular value in the detection and tracking of preclinical ADAD, informing a mutation carrier’s prognosis and evaluating amyloid-β-modifying ADAD treatments.

## Background

Presenilin 1 (PSEN1), PSEN2, and amyloid precursor protein (APP) mutations have been shown to cause autosomal dominant Alzheimer’s disease (ADAD). Because the age of dementia onset is highly predictable in ADAD, studying cognitively unimpaired mutation carriers allows to characterize the temporal sequence of ADAD biomarker changes prior to cognitive decline and to inform the design of ADAD prevention trials. Recent research in families with ADAD supports the hypothesis that amyloid-β (Aβ) plaques accumulate early in the disease process and are followed by extensive tauopathy, neurodegeneration, and progressive cognitive decline [1–4]. Amyloid Pittsburgh compound B (PiB)-PET [5–7] and autopsy [5] data have raised the possibility that some forms of ADAD may be associated with greater fibrillar Aβ deposition in the striatum than the neocortex in the preclinical stages of ADAD, a pattern that is not typically observed in sporadic AD [8]. Because of this regional pattern, we examined whether a striatal PiB biomarker had different predictive value for disease severity, as assessed by tau-PET and memory performance, compared to the most commonly used neocortical PiB biomarker, in cognitively unimpaired PSEN1-E280A mutation carriers and non-carriers from the largest known ADAD kindred due to a single-mutation.

## Methods

### Participants

Data from 34 cognitively unimpaired individuals, living in the metropolitan area of the Aburra Valley in Colombia and descending from a common ancestor, were analyzed. There were 14 carriers of the PSEN1-E280A mutation and 20 non-carriers. The median age of the carriers was 35 years old [28–42]. Carriers in this kindred develop mild cognitive impairment at a mean age of 45 [9] (SD 5.3, range 32–62) [10]. All participants scored a Mini-Mental State Examination (MMSE) of 26 or above and a global Clinical Dementia Rating (CDR) of 0. Twenty-one participants were previously described [3].

### Procedures

Cognitive measures were undertaken at the University of Antioquia (Colombia) and included the MMSE, the CDR, and the word list learning delayed recall memory score from the Consortium to Establish a Registry for Alzheimer Disease (CERAD), validated in a Colombian population [11]. Within 2 months of testing, participants traveled to the Massachusetts General Hospital (MGH) in Boston for brain imaging. MRI was acquired using a Siemens Tim Trio 3-T and PET using a Siemens HR+. Forty- to 60-min dynamic [11C]-Pittsburgh Compound B (PiB; fibrillar Aβ) and 80–100-min [18F]-flortaucipir (FTP; tau) PET scans were acquired, and regional-to-cerebellar gray matter PiB distribution volume ratios (DVRs) and FTP standard uptake volume ratios (SUVRs) were computed as previously described [3]. Freesurfer v.5, co-registered T1-weighted MRIs, and automated regions-of-interest were used to mean neocortical and striatal PiB-DVRs, with no partial volume correction. The neocortical DVRs were generated from bilateral frontal, lateral temporal, and retrosplenial cortices; striatal DVRs were computed from bilateral caudate and putamen. FTP-SUVRs were characterized in the entorhinal cortex ROI, since this region was found to be associated with early FTP-SUVR increases over age in our PSEN1-E280A kindred [3].

Participants provided written informed consent, and approvals were obtained from the University of Antioquia Ethics Committee and the MGH Institutional Review Board. Participants and clinical investigators were blinded to the participants’ mutation status.

### Statistics

*T* tests and *χ*^2^ tests were used to compare the data of mutation carriers and non-carriers. ANOVAs with repeated measures compared PiB-DVRs in the neocortex and striatum in the mutation carriers and non-carriers. Regions-of-interest (neocortex and striatum) were used as repeated factors. Linear regressions estimated the rate of age-related increase in PiB and FTP-PET uptake, and the rate of age-related memory decline. Age-by-group interactions determined the age of PET signal detection, and a 5000-iterations bootstrapping procedure estimated 95th percentile confidence intervals around this age. Spearman’s *R*^2^ were used to characterize the extent to which neocortical and striatal PiB-DVRs were related to entorhinal cortex FTP-SUVRs and CERAD memory scores.

## Results

### Groups’ comparisons

Demographic characteristics, MMSE scores, and CERAD memory scores were not significantly different between mutation carriers and non-carriers. In contrast, entorhinal FTP was higher in the 14 carriers (Table 1), indicating that they had early tau pathology in the absence of detectable cognitive decline.

**Table 1:** Participant characteristics

Mean value *(SD)*	Carriers *N* = 14	Non-carriers *N* = 20	Statistics
Age	35.0	35.8	*t* = − 0.5
Years	*(5.1)*	*(5.0)*	*p* = 0.65
Education	11.1	10.5	*t* = + 0.4
Years	*(4.1)*	*(4.0)*	*p* = 0.69
Female	9	10	*χ*^2^ = 0.7
%	*(64.3%)*	*(50.0%)*	*p* = 0.41
MMSE	28.5	28.9	*t* = − 1.0
	*(1.1)*	*(0.9)*	*p* = 0.34
CERAD	6.6	7.5	*t* = − 1.4
Recall	*(2.3)*	*(1.2)*	*p* = 0.17
Entorhinal	1.24	0.99	***t*** **= + 3.9**
FTP-SUVr	*(0.27)*	*(0.08)*	***p*** **= 0.0005**
Neocortical	1.27	1.02	***t*** **= + 7.1**
PiB-DVR	*(0.15)*	*(0.02)*	***p*** **< 0.0001**
Striatal	1.67	1.24	***t*** **= + 7.7**
PiB-DVR	*(0.25)*	*(0.05)*	***p*** **< 0.0001**

Significant *p*-values are correctly highlighted in bold

Neocortical and striatal PiB-DVRs were higher in mutation carriers than in non-carriers (*p* < 0.0001). Importantly, the between-group difference in PiB uptake (striatum: 0.43DVR between carriers and non-carriers, neocortex: 0.25DVR) was greater in the striatum than in the neocortex (region-by-group interaction: F = 17.1, *p* = 0.0002). Note that the non-carriers also had higher striatal than neocortical uptake (repeated-measure ANOVA: F = 556.4, *p* < 0.0001), presumably reflecting striatal non-specific binding to white matter.

### Relationships to age

**Fig. 1:**
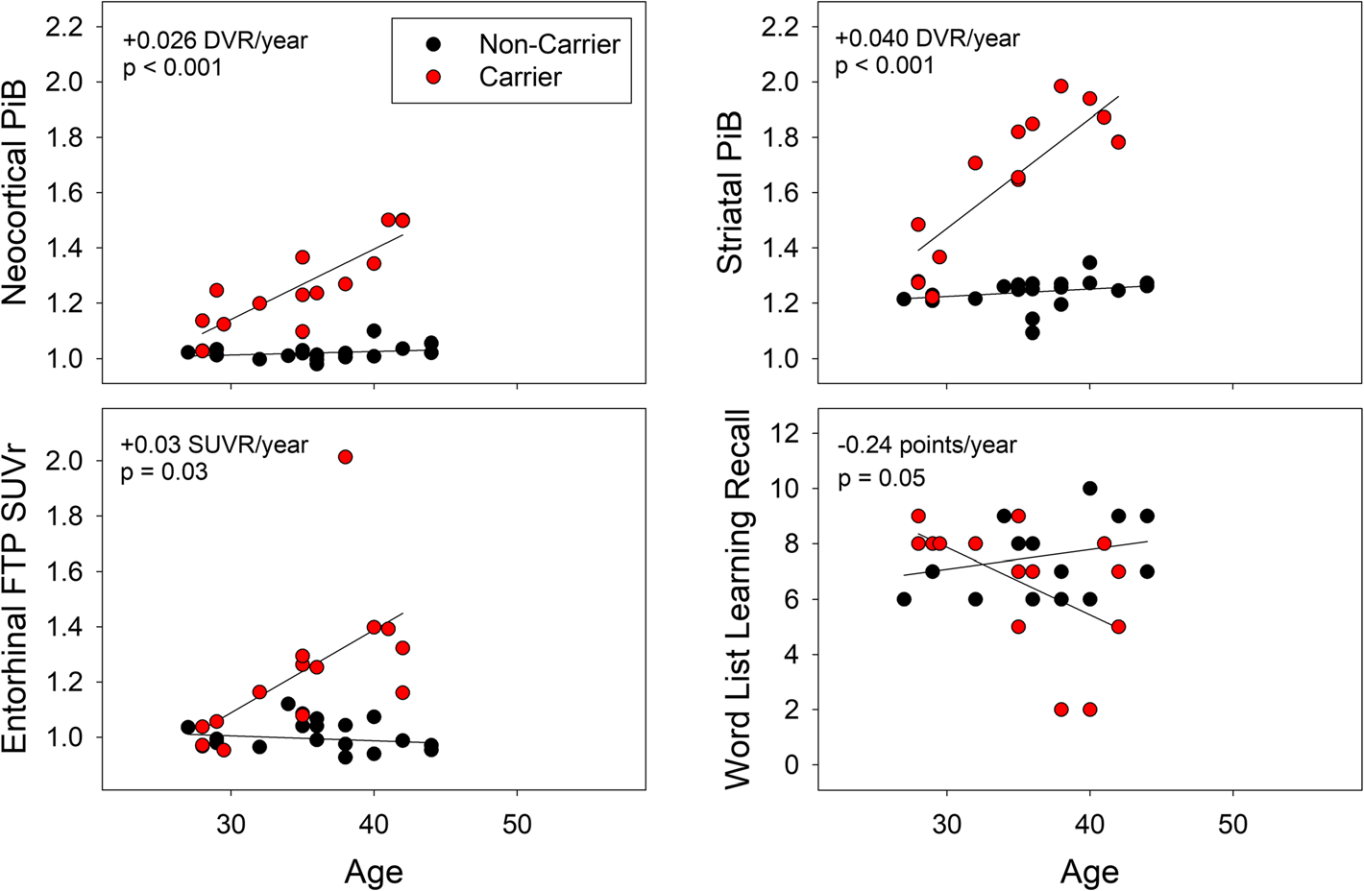
Neocortical and striatal PiB (amyloid), entorhinal FTP (tau), and memory scores as a function of age. Linear regressions estimated the rate of age-related increase in PiB and FTP-PET uptake, and the rate of age-related memory decline in mutation carriers (red) and non-carriers (black). All age-related rates were significantly greater in carriers than non-carriers. The age-related rate in carriers is provided in red font

In the carriers, neocortical and striatal PiB-DVRs, entorhinal FTP-SUVRs, and memory scores were age-related. In the non-carriers, no age relationships were observed (all *p*’s > 0.20). All the age-by-group interactions were significant, such that carriers had greater associations between age and PiB-DVRs (both *p* < 0.0001), entorhinal FTP-SUVRs (*p* = 0.008), and memory scores (*p* = 0.009), than non-carriers. The age-by-group interactions demonstrated that neocortical and striatal PiB-DVRs were higher from age 28 [CI95 27–30], entorhinal FTP-SUVRs were higher from age 32 [30–33], and memory scores were lower in carriers older than 37 [[CI95 34–40] (Fig. 1).

Although PiB increased more with age in the striatum (+ 0.040DVR/year) than in the neocortex (+ 0.026DVR/year), the age relationships were not statistically different between regions (age-by-region interaction in the carriers only: F = 2.0, *p* = 0.18; age-by-region-by-group interaction: F = 2.2, *p* = 0.15).

### Relationships to tauopathy and memory

**Fig. 2:**
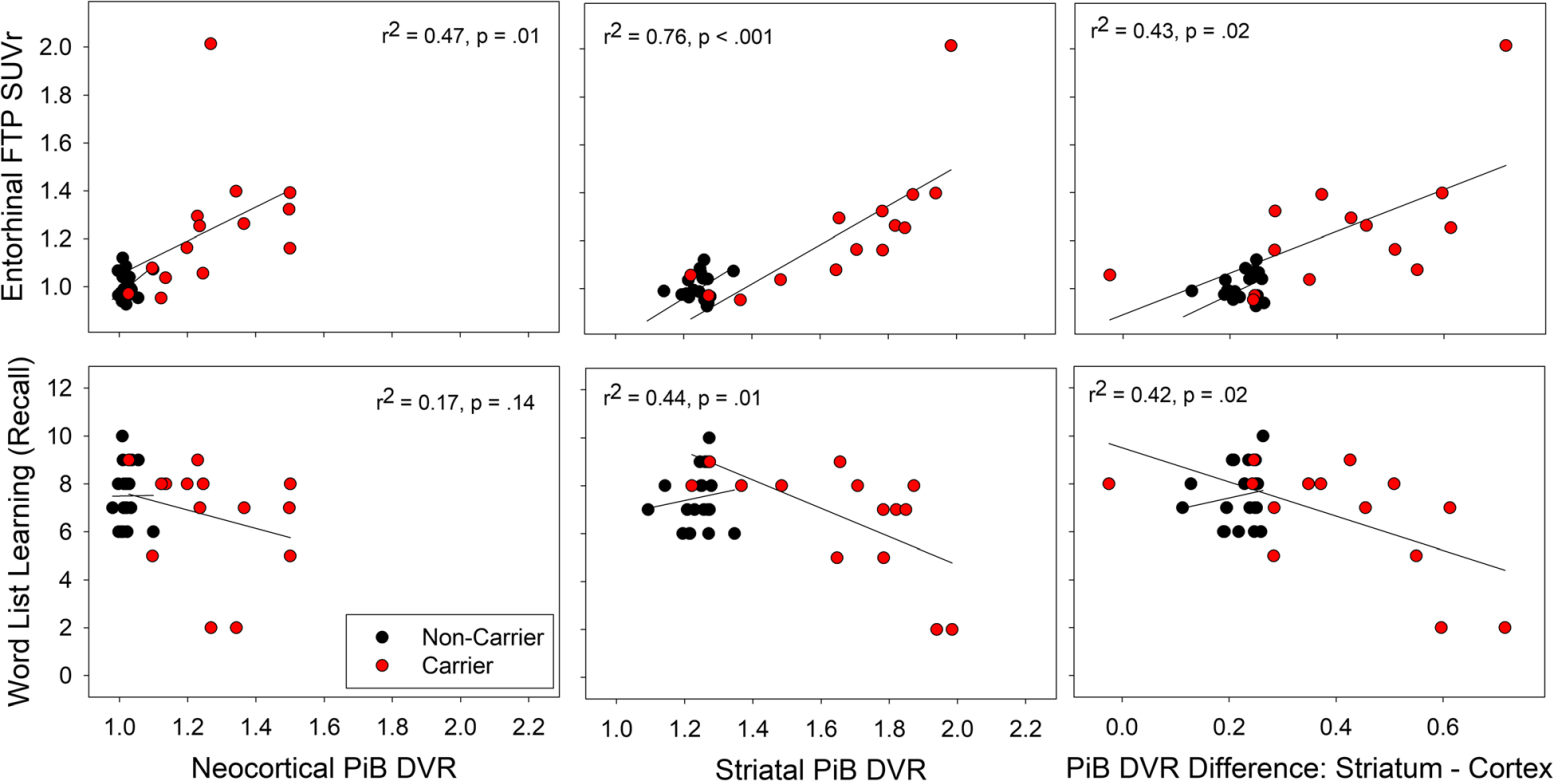
Associations between regional PiB measures, entorhinal FTP, and episodic memory. Spearman’s *R*^2^ were computed between PiB uptake and FTP uptake in the entorhinal cortex (top row) or memory scores (bottom row) in the mutation carriers. In the carriers, significant correlations were observed for all correlations other than the correlation between neocortical PiB-DVR and lower recall memory (*p* = 0.14). There were no significant correlations in the non-carriers. The preferential (striatal>neocortical) association between PiB-DVRs is illustrated on the right. The F-statistics provided is an ANOVA with repeated measures comparing the association of both PiB regions with the outcomes, in the carriers only

In the carriers, neocortical PiB-DVRs were correlated with entorhinal FTP-SUVRs, but not significantly with lower memory scores (Fig. 2—left, *p* = 0.14). In contrast, striatal PiB-DVRs were significantly correlated with both entorhinal FTP-SUVRs and lower memory scores. Striatal PiB-DVRs were distinguished from neocortical PiB-DVRs by significantly greater associations with entorhinal cortex FTP-SUVRs (ANOVA: *p* = 0.02) and lower memory scores (Fig. 2—right, *p* = 0.02). In the non-carriers, neocortical and striatal PiB-DVRs were not significantly correlated with FTP-SUVRs or memory scores (all *R*^2^ < 0.01, *p* > 0.7). The preferential (striatal>neocortical) association between PiB-DVRs and lower memory scores was significantly greater in the carriers than in the non-carriers (F = 5.4, *p* = 0.03).

To test whether higher striatal PiB-DVRs were associated with tauopathy and memory in carriers of the same age, we adjusted the previously observed associations for age and demonstrated that higher striatal PiB-DVRs were associated with higher entorhinal FTP-SUVRs (age-adjusted partial *R*^2^ = 0.50, *p* = 0.007), and lower memory scores (*R*^2^ = 0.28, *p* = 0.09, trend-level), independently of age.

Given the possible confounds of using the cerebellum as reference region, we also compared neocortical and striatal PiB using pons as a reference. Results with pons were very similar to the ones with the cerebellum (e.g., the statistical associations between striatal PiB and entorhinal FTP/memory performance that we observed with cerebellum as reference region were also observed with pons as reference).

## Discussion

In this cross-sectional study, we characterized and compared the extent to which striatal and neocortical PiB-PET measurements of fibrillar Aβ were associated with age, entorhinal tau-PET, and memory scores in cognitively unimpaired Colombian PSEN1-E280A mutation carriers and non-carriers. While striatal and neocortical PiB were both significantly associated with age in the mutation carrier group, the striatal measure was distinguished by significantly greater associations with tau burden and memory scores than the neocortical measure. This study supports the value of striatal PiB-PET measurements to predict disease severity in the preclinical stage of ADAD.

The association of striatal PiB with entorhinal FTP and memory scores survived age-adjustment, indicating that striatal Aβ deposition could potentially be used to predict short-term clinical progression in carriers of the same age. Longitudinal and treatment studies are ongoing to compare striatal and neocortical PiB measurements in their ability to track Aβ plaque deposition, evaluate Aβ treatments, and provide prognostic information in cognitively unimpaired ADAD mutation carriers.

Additional studies are needed to clarify the extent to which these findings are relevant to other Aβ-PET tracers, which may be less sensitive to the detection of preclinical and particularly diffuse Aβ plaques. Unlike [11C]-PiB observations in other ADAD families [5–7], a previous study of the Colombian kindred observed similar age-related Aβ deposition in the cortex and striatum, using [18F]-florbetapir [2]. The mechanisms leading to early elevations in striatal PiB-PET in ADAD are not fully elucidated, but higher affinity of [11C]-PiB for diffuse plaques, predominantly observed in the striatum, as opposed to neuritic plaques that are more often seen in the neocortex [5], was suggested as a potential mechanism for the discrepant results obtained with the different tracers. Higher striatal non-specific binding with florbetapir might also account for a lower sensitivity [8]. However, case reports [12] indicate that [18F]-compounds are able to visualize early striatal Aβ in ADAD. Using PiB, we observed higher Aβ burden in the striatum than in the neocortex, and the rate of age-related Aβ deposition was nominally greater in the striatum, although not significantly greater, than in the neocortex. In the Colombian kindred, Aβ deposits thus in both regions, with a marginally faster rate in the striatum. Besides the tracers used, ADAD studies also differ in the mutations responsible for the disease [6], and regional Aβ deposition might be dependent on the specific mutation type. A previous study including carriers of different PSEN1 mutations indeed observed regional heterogeneity [13]. Further research, including studies from the Dominantly Inherited Alzheimer’s Network (DIAN), could help clarify the extent to which findings in PSEN1-E280A mutation carriers are relevant to those with other mutations. Recent data suggest that, on average, individuals included in the DIAN study demonstrate amyloid accumulation in precuneus before the striatum [14]. However, the relations of amyloid in both regions with tauopathy and memory performance still require evaluation.

## Conclusions

In this cross-sectional study of the Colombian kindred, striatal PiB-PET measurements better correlated with tau-PET measurements and memory performances than neocortical PiB-PET measurements. Future studies will evaluate the value of striatal PiB-PET to track Aβ accumulation in longitudinal preclinical ADAD studies, to inform a mutation carrier’s prognosis, and to evaluate Aβ-modifying ADAD treatments.

### Consent

Written informed consents were obtained from all the participants for the publication of this manuscript. Copies of the written consents are available for review by the Editor-in-Chief of this journal. Approvals were obtained from the University of Antioquia Ethics Committee and the MGH Institutional Review Board before that participants underwent any procedures.

## Abbreviations

ADAD: Autosomal dominant Alzheimer’s disease
APP: Amyloid precursor protein
Aβ: Amyloid-β
CDR: Clinical Dementia Rating
CERAD: Consortium to establish a registry for Alzheimer’s disease
DVR: Distribution volume ratio
FTP: Flortaucipir
MGH: Massachusetts General Hospital
MMSE: Mini-Mental State Examination
PiB: Pittsburgh Compound B
PSEN: Presenilin
SUVr: Standard uptake volume ratio
